# Supplementary material for: Febrile-Range Hyperthermia Can Prevent Toxic Effects of Neutrophil Extracellular Traps on Mesenchymal Stem Cells
Source: Int J Mol Sci. 2022 Dec 19;23(24):16208. doi: 10.3390/ijms232416208 (PMC9786713; doi:10.3390/ijms232416208)
Supplement: Supplementary file 1 [file ijms-23-16208-s001.zip › ijms-2008147-supplementary.pdf]

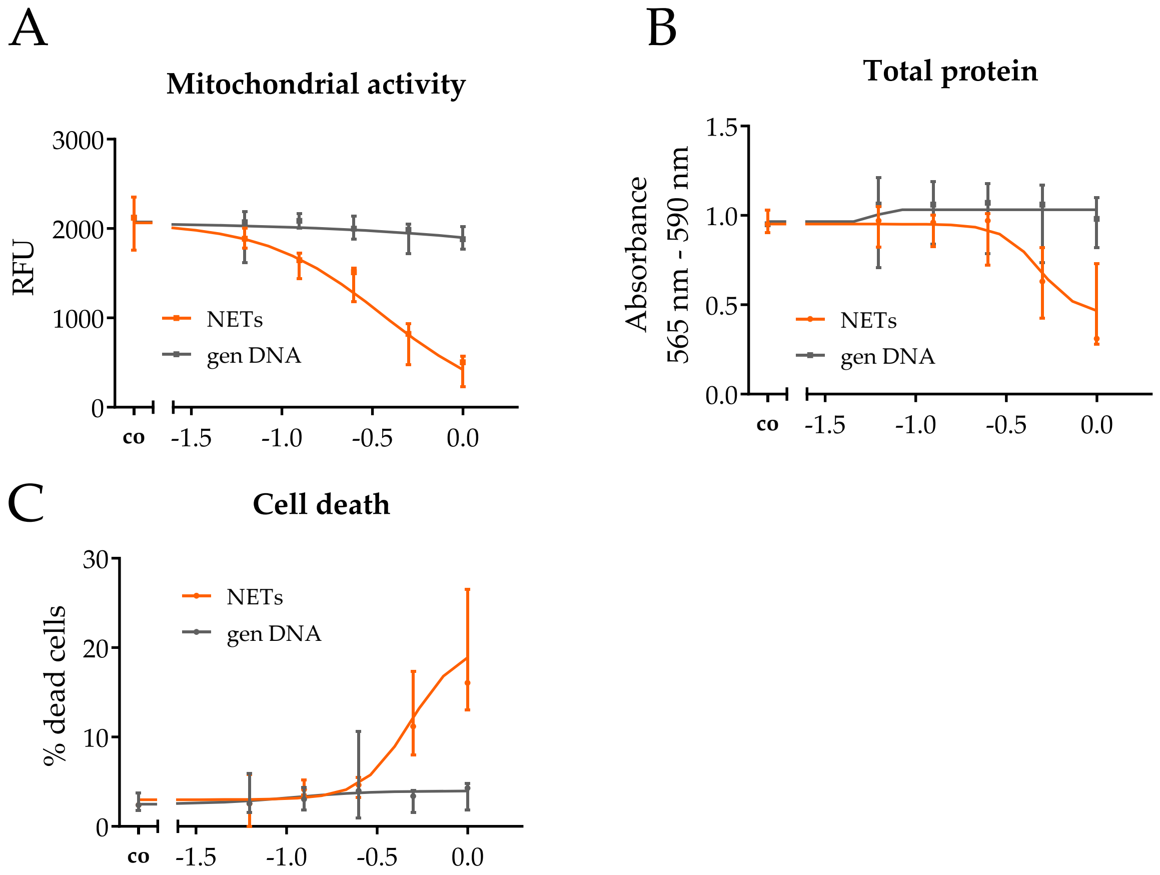

Supplementary Figure S1: Calculation of EC50 values from **(A)** mitochondrial activity (resazurin conversion) values **(B)** total protein content (SRB staining) and **(C)** LDH release as indicator for cell death (in relation to lysed cells). Values were plotted against the log10 of NETs concentration in ng/µL and EC50 calculcated with Graphpad Prisma 8. N=3, n=3.

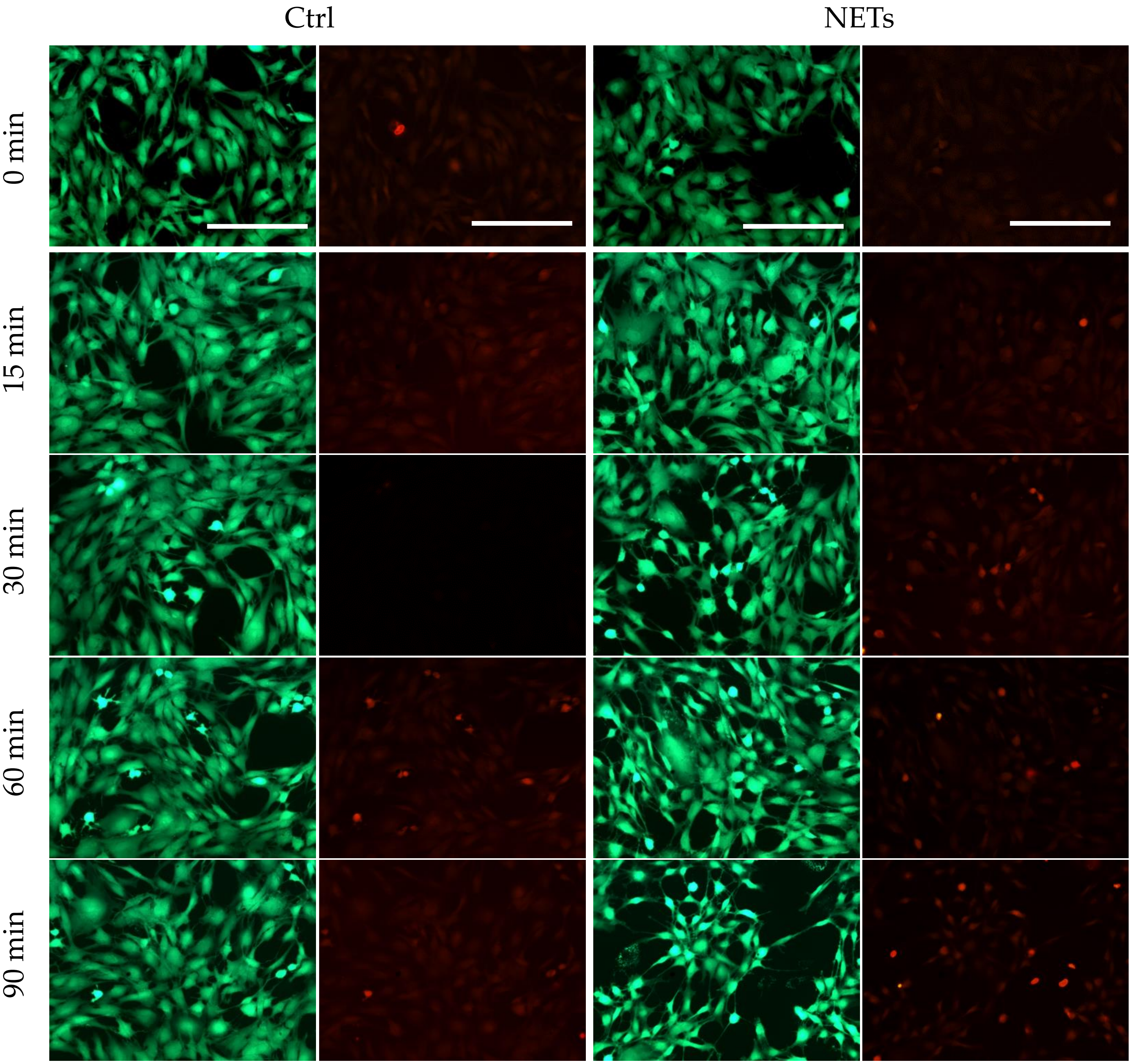

Supplementary Figure S2: Incubation of SCP-1 cells with NETs in PBS. SCP-1 cells were pre-incubated with Calcein AM for 1 h, washed twice with PBS and propidium iodide (and 0.5 ng/µL NETs) added. Microscopy was done at the indicated time points. Scale bar 200 µm.

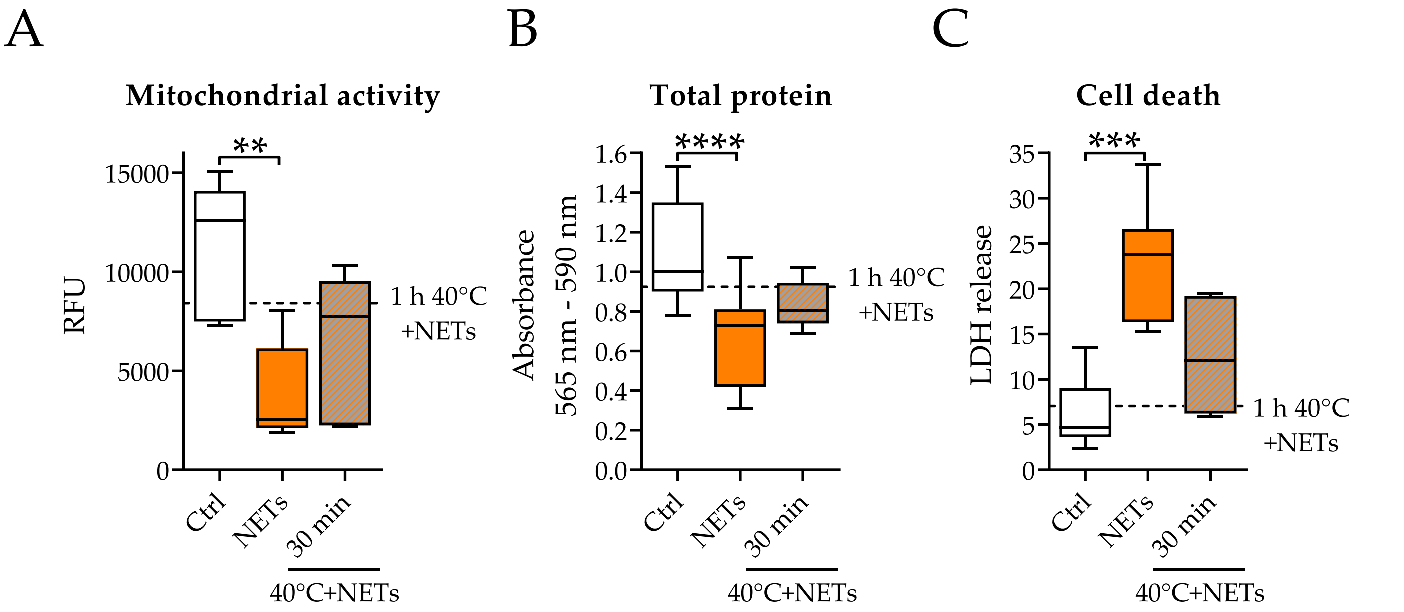

Supplementary Figure S3: Effect of 30 min denaturation of NETs at 40°C **(A)** mitochondrial activity (resazurin conversion) values **(B)** total protein content (SRB staining) and **(C)** percentage of dead cells (LDH release in relation to lysed cells). Dashed line indicates mean values of 1 h treatment at 40°C. **p<0.01, ***p<0.001, ****p<0.0001 as determined by the Kruskal-Wallis test. N=3, n=3.

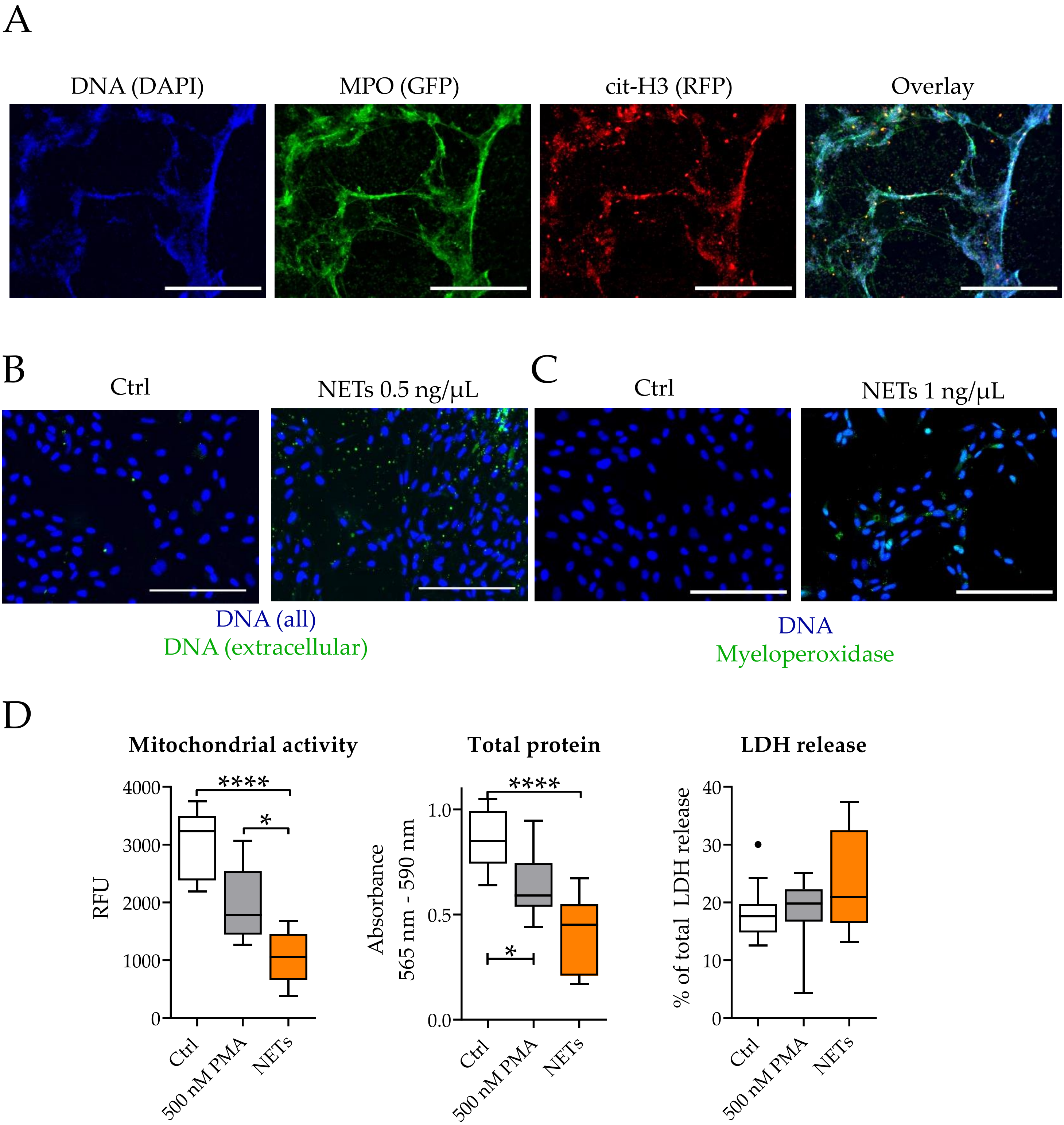

Supplementary Figure S4: Verification of NET formation and further analysis of NETs on MSCs **(A)** Neutrophils treated with 500 nM PMA for 4 h, fixed with 4% formaldehyde and stained for DNA (Hoechst 33342, blue) myeloperoxidase (green) and citrullinated histone 3 (red). Scale bar 400 µm.**(B)** SCP-1 cells (± 0.5 ng/µL NETs) were stained with 1 µM Hoechst and 1 µM Sytox Green **(C)** SCP-1 cells (± 0.5 ng/µL NETs) were fixed with 4% formaldehyde and stained for DNA (Hoechst 33342, blue) and myeloperoxidase (green). Antibodies: myeloperoxidase: 1:200, sc-52707, Santa Cruz Biotechnology; citrullinated histone 3: 1:100, #5103, Abcam **(D)** Effect of PMA on SCP-1 cells. SCP-1 cells were incubated with 500 nM PMA for 48 h and mitochondrial activity (resazurin conversion, left) total protein content (SRB staining, middle) and (C) LDH release (in relation to lysed cells, right) measured. N=3, n=4. *p<0.05, **p<0.01, ***p<0.001, ****p<0.0001 as determined by the Kruskal-Wallis test.
